# Management of Different Types of Root Resorption Affecting Traumatised Permanent Maxillary Incisors—A Case Report

**DOI:** 10.1155/crid/6262904

**Published:** 2025-10-29

**Authors:** Manisha Deepanjan, Sharan S. Sargod, K. Sundeep Hegde, H. T. Ajay Rao

**Affiliations:** Department of Pediatric and Preventive Dentistry, Yenepoya (Deemed to Be University) Dental College, Mangaluru, Karnataka, India

**Keywords:** bioceramic materials, external root resorption, pulp calcification, tooth injuries

## Abstract

Managing defects caused by pathological root resorption in permanent teeth can be challenging. If not diagnosed and treated efficiently, progressive root resorption can result in the loss of a tooth. This paper reports the consequences and treatment of poorly managed dental trauma 3 years ago affecting maxillary central and lateral incisors of an 11-year-old male child. Intraoral periapical radiographs revealed resorption defects involving the cervical, middle and apical thirds of Tooth Numbers 12, 11, 21 and 22 (FDI numbering system). A blunderbuss canal with a wide open apex was also noted in relation to Tooth Number 22. Resorption defects involving the pulp canal space and calcification of the pulp were also noted in relation to Tooth Numbers 21 and 12, respectively. This was confirmed by a CBCT scan. A diagnosis of infection-related root resorption, invasive cervical root resorption with pulp canal calcification and internal root resorption was made in relation to Tooth Numbers 11, 22, 12 and 21, respectively. The cervical defect in relation to Tooth Number 22 was surgically debrided and filled using MTA. Nonsurgical endodontic treatment was performed in relation to Tooth Numbers 11 and 21, and apexification was performed in Tooth Number 22. Bioceramic materials like MTA and Biodentine were used to seal the resorption defects as well as obturate the entire root canals of the affected teeth. On 36 months' follow-up, the treated teeth were clinically asymptomatic, showed healing radiographically, and there was no further progression of root resorption. The outcome of this case emphasises the importance of timely management and appropriate clinical care in all traumatic dental injuries. The role of bioceramics in managing root resorptions seems to be promising.

## 1. Introduction

Root resorption in permanent teeth is a sequelae following dental trauma. It is a pathological process and results in the destruction of cementum and dentin. If left untreated, it could lead to loss of the affected teeth [1, 2].

The roots of permanent teeth are resistant to resorption as the precementum and predentin layers exhibit protective effects on the root surface. The outermost aspect of cementum is enveloped by a zone of nonmineralised cementoid matrix, and the innermost aspect of dentin is covered by predentin matrix [3, 4].

Clastic cells do not resorb or adhere to unmineralised matrix. RGD (arginyl-glycyl-aspartic acid) peptides are the mediators of osteoclast binding, and these are present only on mineralised surfaces. The cemental layer also prevents the passage of toxins from the infected root canal into the surrounding periodontal tissues [5, 6]. Loss or alteration of these protective layers can occur due to trauma or indirectly due to an inflammatory response to a traumatic injury [2, 7].

Resorption of the root can present in different forms, such as internal and external resorption or both, depending on the surface affected. Internal root resorption affects the internal aspect of the root and occurs only if the predentin and odontoblastic layers are altered or lost. External root resorption affects the outer surface of the root, and it could be cervical, inflammatory, replacement or surface resorption [8]. External inflammatory root resorption (EIRR) is now referred to as infection-related root resorption (IRR) [9]. External root resorption is more common as compared to internal root resorption. Invasive cervical root resorption is an aggressive form of external root resorption, and it affects the cervical third of the root [10]. Pulp necrosis can also arrest the development of the root in immature teeth, resulting in a blunderbuss canal with an open apex.

This case report describes the management of teeth affected by IRR, internal root resorption, cervical root resorption with pulp canal calcification and open apex using bioceramic materials. The report is in accordance with the PRICE 2020 guidelines. The patient and his legal guardian were informed about the report, and they agreed to participate, signing an informed consent.

## 2. Case Report

An 11-year-5-month-old Indian male patient reported to the Department of Pediatric and Preventive Dentistry, Yenepoya Dental College, Mangalore, with a complaint of pain and mobility of the left upper front tooth since 1 month. There was no contributory medical history.

According to the parent, the child suffered a trauma that occurred approximately 3 years ago to upper central and lateral incisors, resulting in the fracture of Tooth Numbers 21 and 22 (FDI tooth numbering system), displacement and mobility of Tooth Numbers 11, 21, 22 and 22, indicating lateral luxation. The patient had visited a primary healthcare centre for emergency care. Lacerated gingiva and lips were sutured. The patient consulted a local dentist only after 10 days as the mobility of the affected teeth did not subside. The dentist then splinted the mobile teeth for approximately 4 weeks and performed root canal treatment of Tooth Number 11, followed by the restoration of Tooth Numbers 11, 21 and 22. Furthermore, the father reported that clinical and radiographic follow-up was not performed after the treatment.

**Figure 1:**
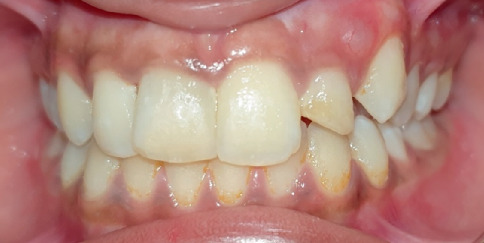
Pre-treatment intraoral clinical photograph.

Clinical examination revealed discoloured Tooth Numbers 12, 11, 21 and 22 (Figure 1). Tooth Numbers 11 and 21 presented with fractured restorations and Grade I mobility. Tooth Number 22 was tender on percussion and presented with Grade II mobility and a sinus tract. A periodontal pocket of 8 mm was noted in relation to the distal aspect of Tooth Number 12, with no signs of gingival inflammation and mobility. There was no occlusal interference of the affected teeth with the opposing dentition.

Pulp vitality tests were negative in relation to Tooth Numbers 11, 12, 21 and 22 and were contrasted with the normal responses of the adjacent unaffected teeth.

**Figure 2:**
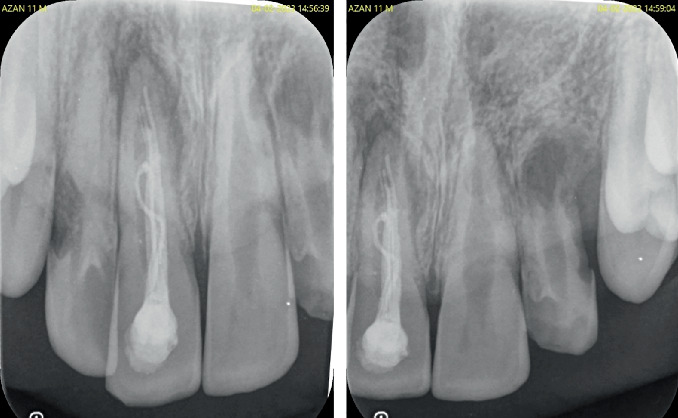
Pre-treatment intraoral periapical radiographs in relation to Tooth Numbers 12, 11, 21 and 22.

Intraoral periapical radiographic examination revealed the following (Figure 2):

a. Tooth Number 12—Ill-defined radiolucency involving the cervical aspect of the radicular surface and obliterated pulp canal space.
b. Tooth Number 11—Ill-defined radiolucencies involving the middle and apical thirds of the radicular surface and incomplete root canal treatment.
c. Tooth Number 21—Ill-defined radiolucencies involving the middle and apical thirds of the radicular surface.
d. Tooth Number 22—Blunderbuss canal, open apex with apical radiolucency and ill-defined radiolucencies along the radicular surface.

**Figure 3:**
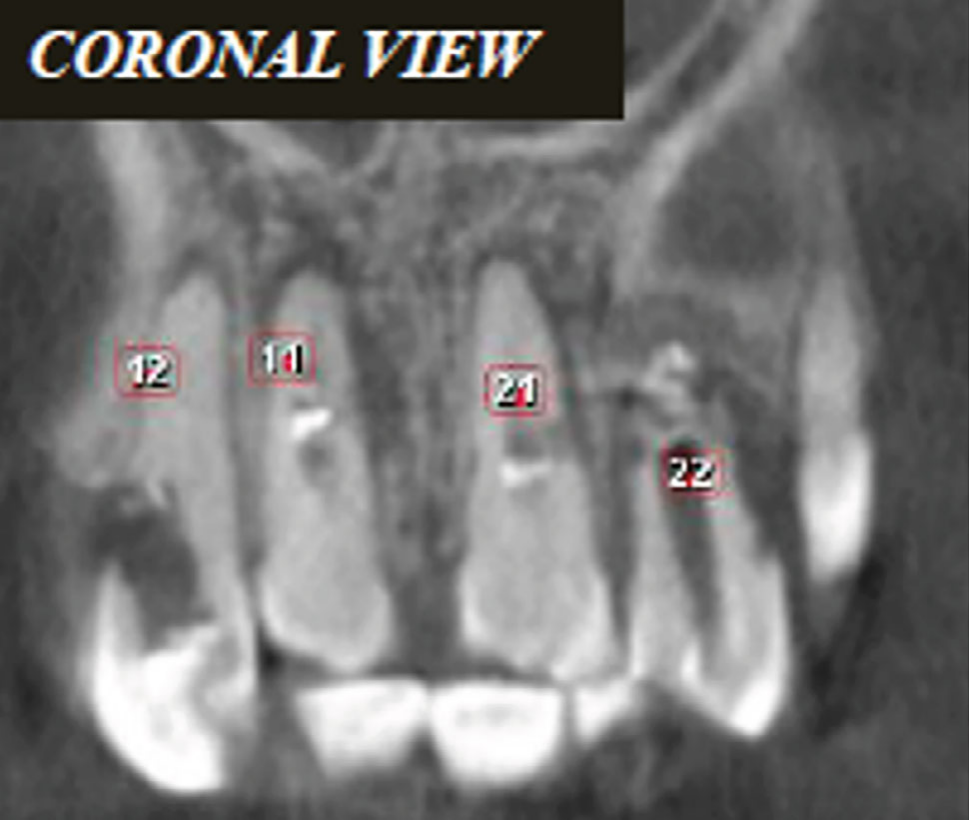
CBCT image showing Tooth Numbers 12, 11, 21 and 22.

Cone beam computed tomography (CBCT) (Planmeca Romexis, Finland) was done to aid in the diagnosis (Figure 3).

**Figure 4:**
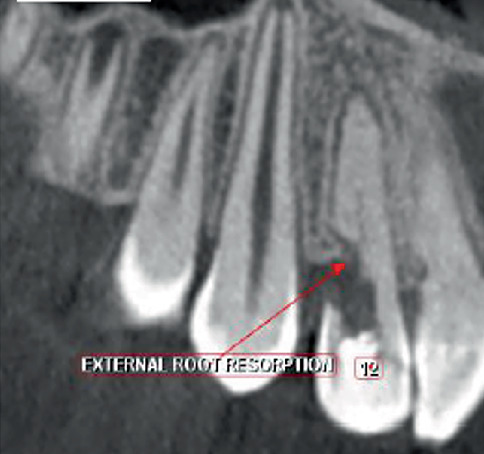
CBCT image showing coronal view of Tooth Number 12 with cervical root resorption and calcified root canal.

**Figure 5:**
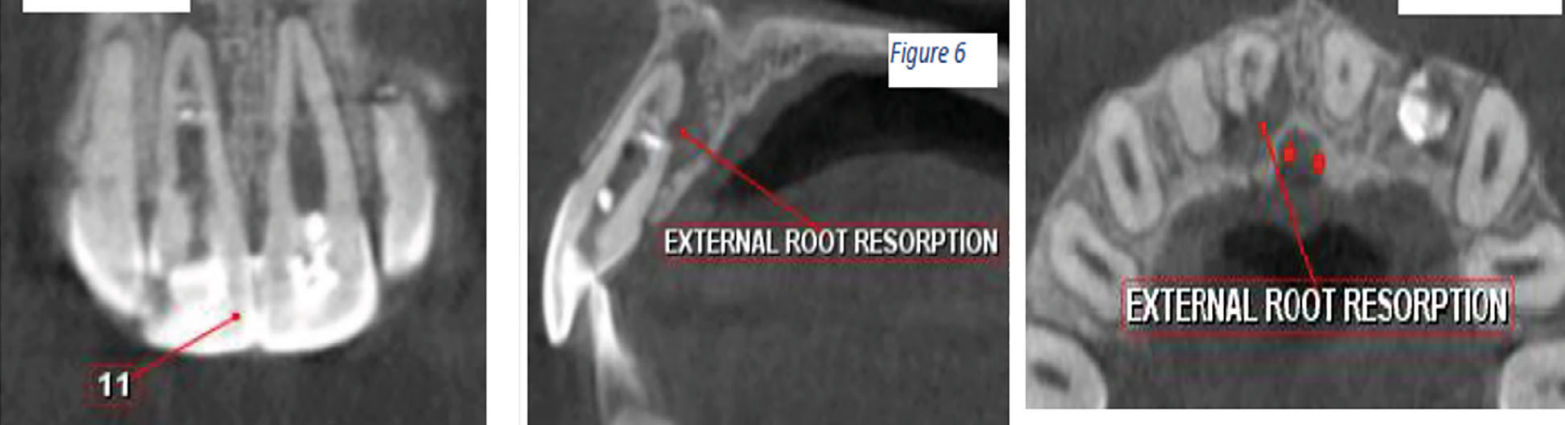
CBCT images showing coronal, sagittal and axial view of Tooth Number 11 with external root resorption.

**Figure 6:**
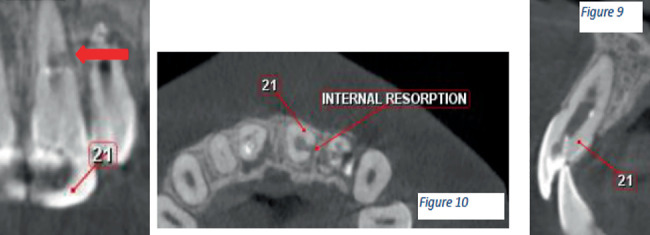
CBCT images showing coronal, axial and sagittal views of Tooth Number 21 with internal root resorption.

**Figure 7:**
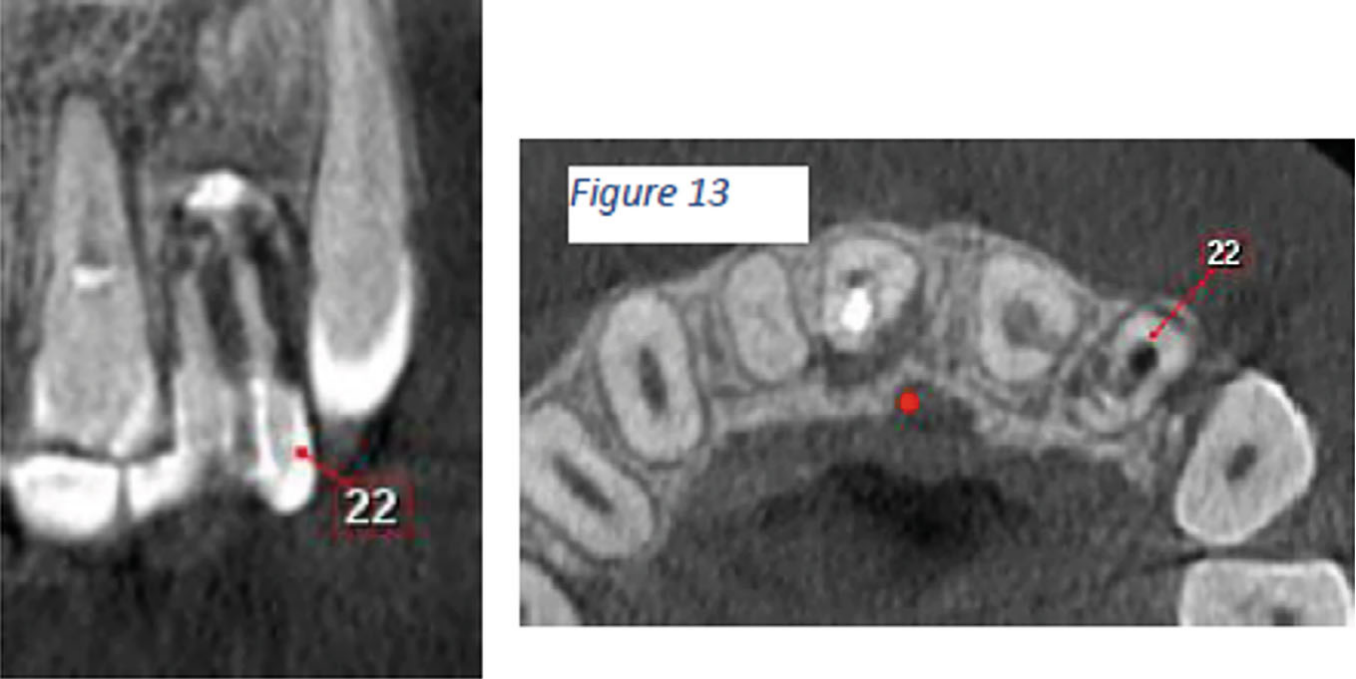
CBCT images showing coronal and axial views of Tooth Number 22 with external root resorption and blunderbuss canal with open apex.

CBCT images revealed the following:

1. Tooth Number 12—Coronal view revealed irregularly shaped hypodensity at the cervical third of the root causing discontinuity of the distal aspect of the root suggestive of cervical root resorption. Root canal space can be noted only up to the cervical third of the root, suggestive of calcified root canal (Figure 4).
2. Tooth Number 11—Coronal, sagittal and axial views revealed an irregularly shaped hypodensity at the palatal aspect of the apical one-third of the root suggestive of external root resorption (Figure 5).
3. Tooth Number 21—Coronal, sagittal and axial views revealed the root canal appears to be widened in the middle third of the root, and an ill-defined hypodensity is noted extending from the canal space towards the distal root surface at the level of the middle third of the root, suggestive of internal root resorption (Figure 6).
4. Tooth Number 22—Coronal and axial views revealed a blunderbuss canal, an open apex with periapical radiolucency and associated with external root resorption involving the middle and cervical thirds of the root (Figure 7).

### 2.1. Diagnosis

With the aid of intraoral periapical radiographs and CBCT images, the following diagnoses were made:

1. Tooth Number 12—Invasive cervical root resorption and pulp canal calcification with apical periodontitis.
2. Tooth Number 11—IRR with apical periodontitis.
3. Tooth Number 21—Internal inflammatory root resorption with apical periodontitis.
4. Tooth Number 22—Blunderbuss canal, open apex with IRR and apical periodontitis.

### 2.2. Treatment Plan

Treatment plan included the following:

a. Re-endodontic treatment in relation to Tooth Number 11.
b. Endodontic treatment in relation to Tooth Number 21.
c. Surgical repair and restoration of the cervical resorptive defect in relation to Tooth Number 12.
d. Endodontic treatment and apexification in relation to Tooth Number 22.

The patient and his legal guardian were informed about the treatment procedures and the follow-up evaluations. Written informed consent was obtained before the initiation of the treatment.

### 2.3. Management of Tooth Numbers 11 and 21

In the first appointment, local anaesthesia was not administered as the affected teeth were nonvital. Rubber dam placement was avoided due to the mobility of the traumatised teeth. To maintain asepsis, the teeth were isolated using cotton rolls and high suction volume.

**Figure 8:**
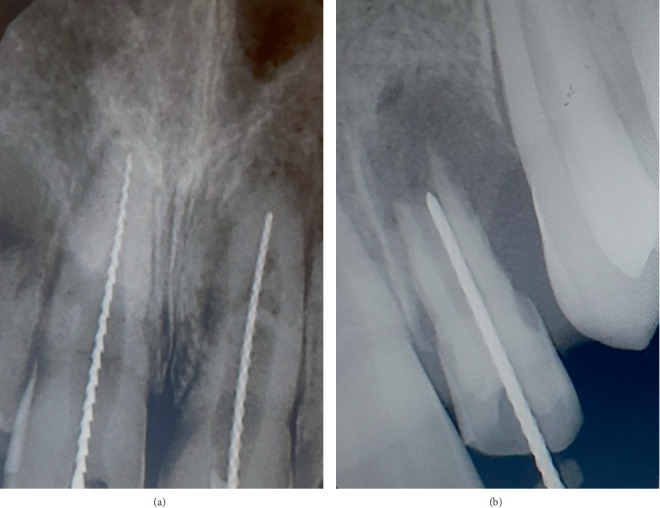
Radiographs of estimation of working length in relation to Tooth Numbers (a) 11 and 21 and (b) 22.

Coronal access was done using an Endo Access bur (Dentsply Maillefer, United States) and an Endo Z bur (Dentsply Maillefer, United States) in relation to Tooth Numbers 11 and 21. Gutta-percha in relation to 11 was removed using Hedstrom files (Mani Inc., Tochigi Ken, Japan). Working length determination was done using an apex locator and verified with radiographs (Figure 8a).

Chemomechanical preparation of the canal was performed using K files (Mani Inc., Tochigi Ken, Japan) up to size #80. The canals were irrigated using 2.5% sodium hypochlorite and saline under agitation. Calcium hydroxide was placed in the root canal as an intracanal medicament, and the cavity was temporarily sealed.

**Figure 9:**
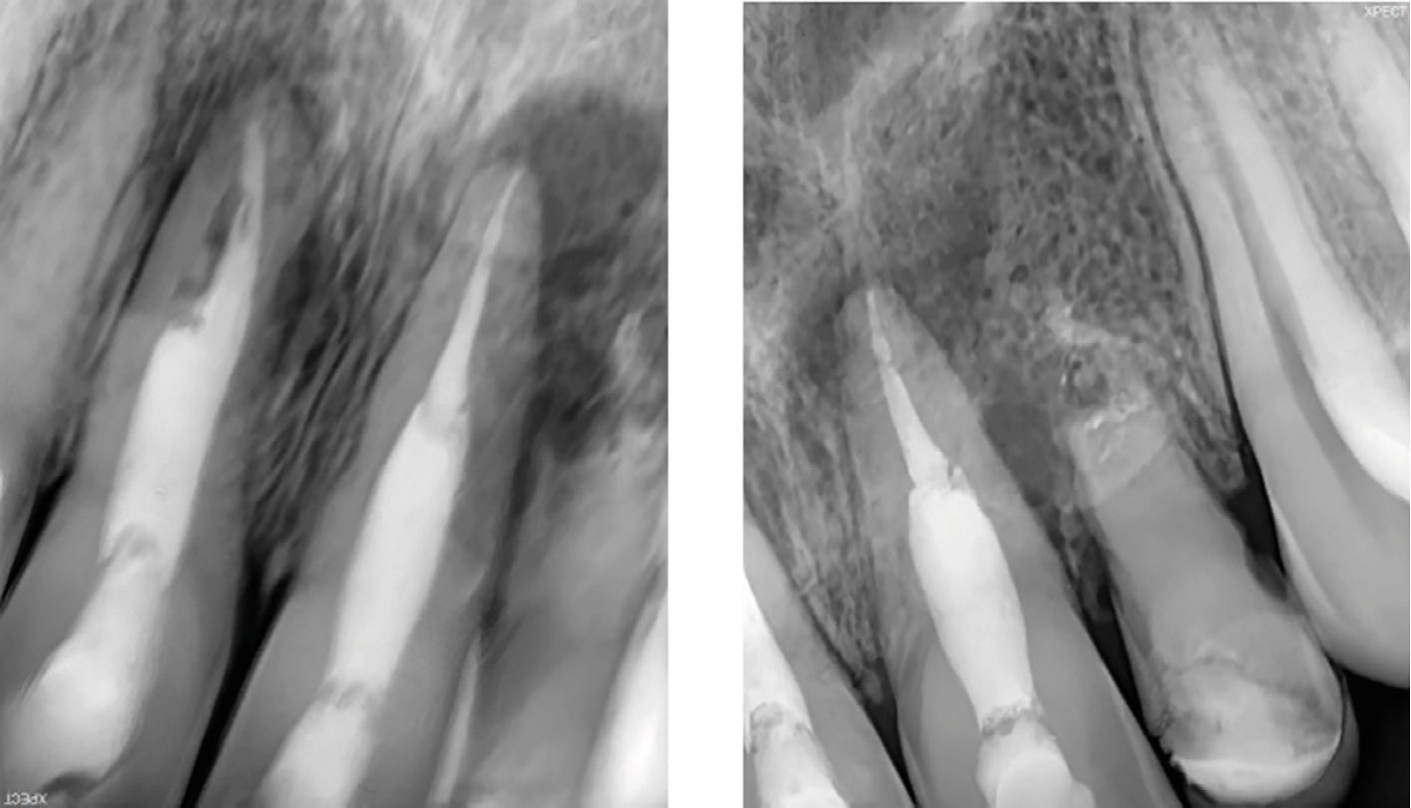
Radiographic images of obturation done in relation to Tooth Numbers 11 and 21 using MTA and in Tooth Number 22 using Biodentine.

In the second visit after 2 weeks, the intracanal medicament was removed by irrigation using saline. The canals were dried with absorbent paper points. Premixed injectable mineral trioxide aggregate (MTA) (Maruchi Endocem MTA, United States) was used to obturate the entire canals of Tooth Numbers 11 and 21 (Figure 9). The access cavities were then restored with glass ionomer cement (GC Fuji IX Extra; GC Co., Tokyo, Japan). No occlusal reduction was performed as there was no occlusal interference with the opposing dentition.

On 6 months follow-up, healing of apical radiolucency was noted, and the mobility resolved to Grade 0.

### 2.4. Management of Tooth Number 22

In the first appointment, local anaesthesia (Lignox, Indoco Remedies Ltd., India) was administered. Rubber dam placement was avoided due to the mobility of the traumatised tooth. To maintain asepsis, the teeth were isolated using cotton rolls and high suction volume.

Access to the pulp chamber was done using Endo Access bur (Dentsply Maillefer, United States). Working length determination was done using No. #80 K file (Mani Inc., Tochigi Ken, Japan) and was kept 1 mm short of the apex (Figure 8b). The debridement of the canal was done by minimal instrumentation and by irrigation using 2.5% sodium hypochlorite, saline and 2% chlorhexidine. Calcium hydroxide dressings were changed once every 2–3 weeks until the healing and resolution of apical pathology were satisfactory.

In the subsequent appointment, the intracanal medicament was removed by flushing the canal with 2.5% sodium hypochlorite and saline. The canal was dried using sterile paper points. Biodentine (Septodont, India) was used in increments to create an apical barrier and obturate the entire length of the root canal of Tooth Number 22 (Figure 9). The access cavity was then sealed using glass ionomer cement (GC Fuji IX Extra; GC Co., Tokyo, Japan). No occlusal reduction was performed as there was no occlusal interference with the opposing dentition.

On 6 months' follow-up, healing of apical radiolucency was noted. At follow-up visits, Tooth Number 22 demonstrated progressive improvement in mobility. On 6 months' follow-up, the mobility resolved to Grade I, and on 12 months' follow-up, the mobility resolved to Grade 0.

### 2.5. Management of Tooth Number 12

Even though the intraoral periapical radiograph and CBCT images had shown obliterated root canal involvement in the middle and apical thirds of the root, an attempt was made to gain access to the canal space in relation to Tooth Number 12.

**Figure 10:**
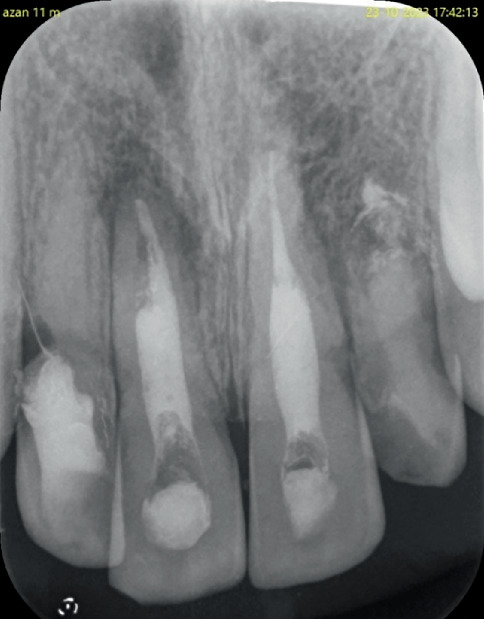
Radiographic image of separated file fragment in cervical defect and obliterated canal space in relation to Tooth Number 12.

Access opening was done using Endo Access bur (Dentsply Maillefer, United States). A size 10 K file was used to check the patency and negotiate the root canal. In the process, the instrument got separated and lodged in the cervical defect and calcified canal space (Figure 10). The attempt to retrieve the broken file was futile; therefore, surgical repair was performed.

**Figure 11:**
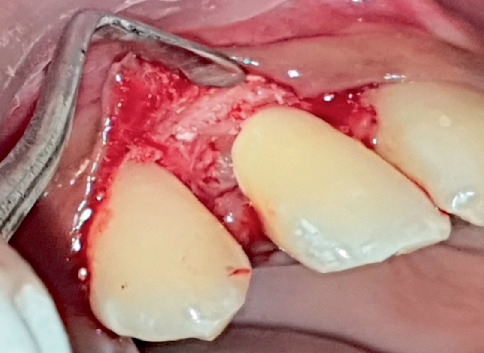
Clinical photograph of the resorption defect on the distal aspect of the cervical third of the root in relation to Tooth Number 12.

**Figure 12:**
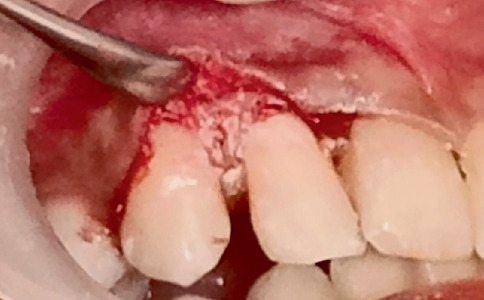
Clinical photograph of the filling of the defect using MTA after debridement of the granulation tissue in relation to Tooth Number 12.

**Figure 13:**
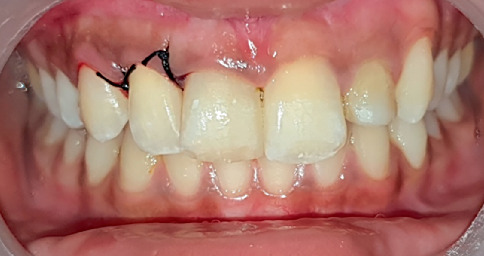
Clinical photograph of flap repositioning and suturing in relation to Tooth Number 12.

**Figure 14:**
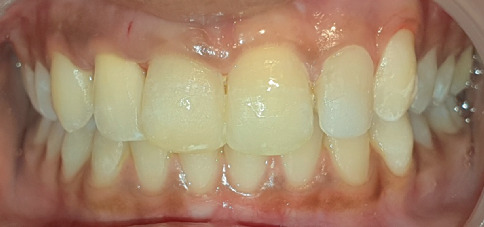
Post-treatment intraoral clinical photograph.

After local anaesthesia, a sulcular incision was given in relation to Tooth Number 12, and a mucoperiosteal flap was reflected. The perforation defect was noted on the distal aspect of the cervical third of the root (Figure 11). The separated file fragment was removed. The granulation tissue was curetted and was cleaned using saline. The defect was then filled with premixed MTA (Maruchi Endocem MTA, United States) (Figure 12). The flap was repositioned and sutured (Figure 13). Post-endodontic restoration was done in relation to Tooth Number 22 using composite (Tetric N-Ceram, Ivoclar) (Figure 14).

**Figure 15:**
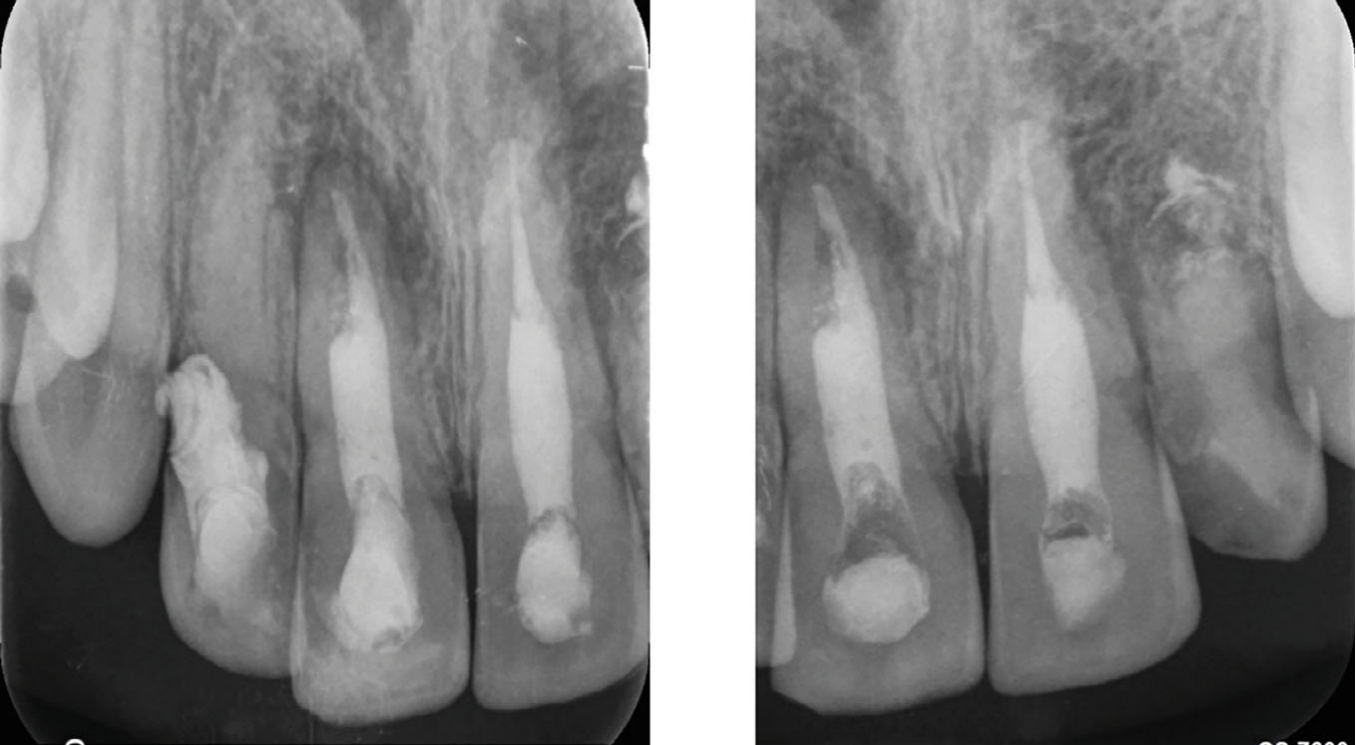
Intraoral periapical radiographs of 6 months' follow-up.

**Figure 16:**
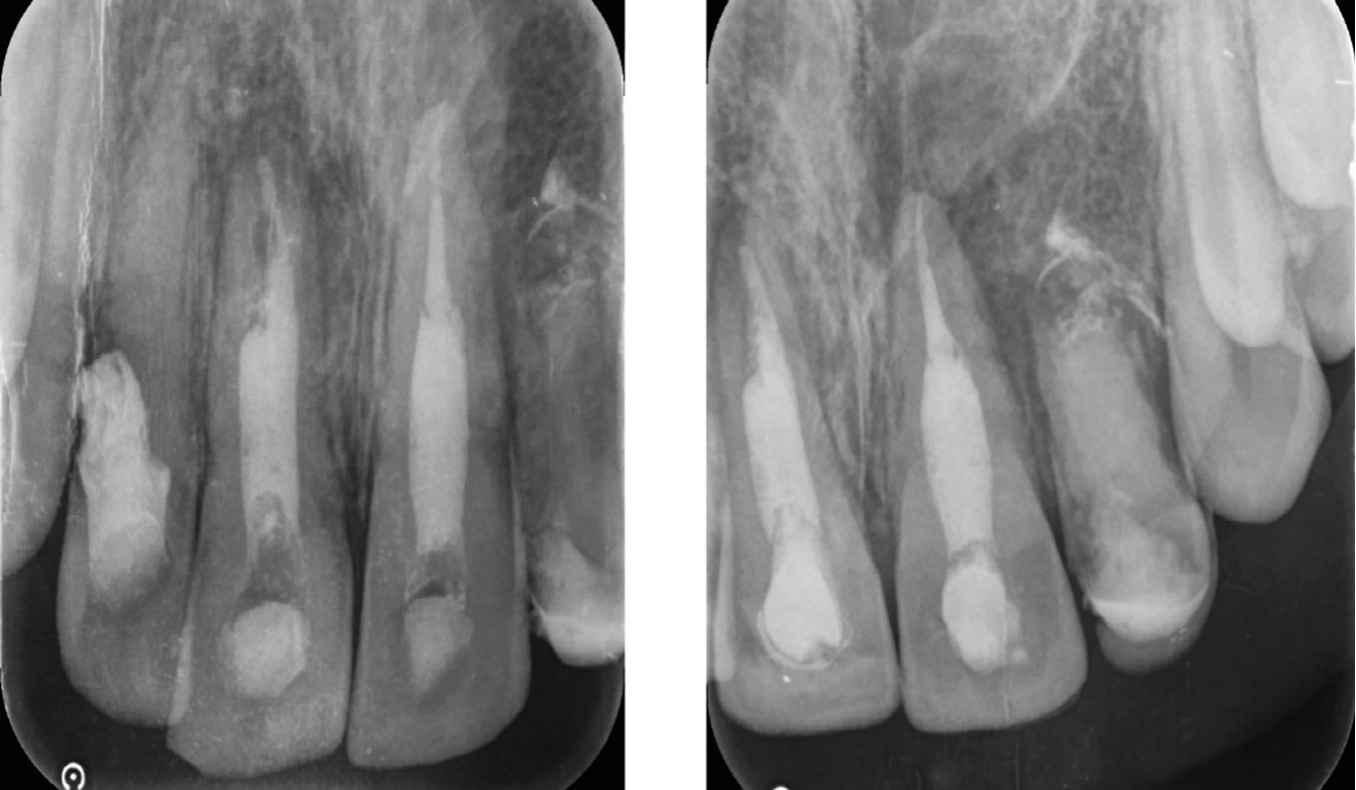
Intraoral periapical radiographs of 12 months' follow-up.

**Figure 17:**
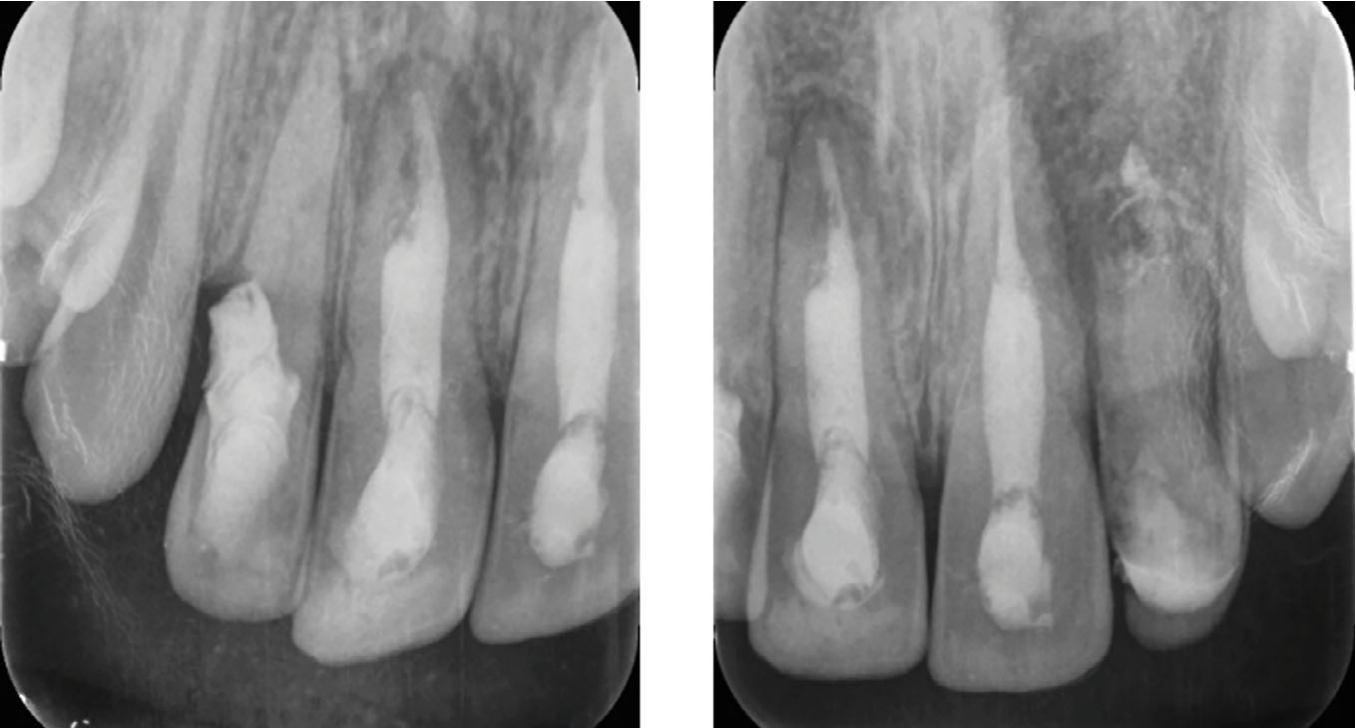
Intraoral periapical radiographs of 24 months' follow-up.

**Figure 18:**
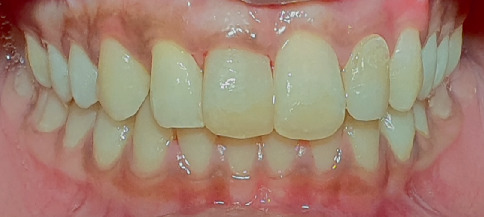
Intraoral clinical photograph of 36 months' follow-up.

**Figure 19:**
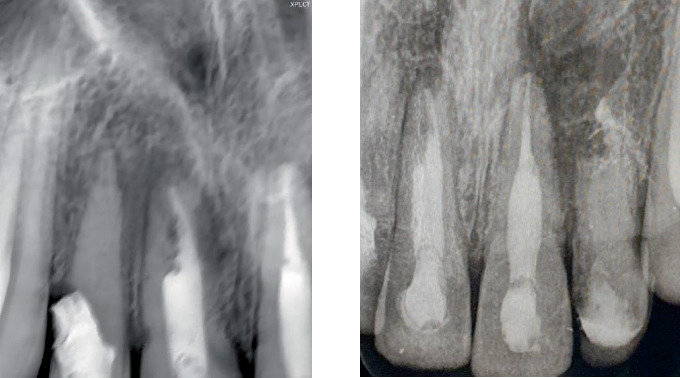
Intraoral periapical radiographs of 36 months' follow-up.

During the follow-up visits, intraoral periapical radiographs were recorded at 6 months (Figure 15), 12 months (Figure 16), 24 months (Figure 17) and 36 months (Figures 18 and 19). The treated teeth were clinically asymptomatic and showed healing radiographically.

The patient expressed satisfaction and comfort with the treatment process, stating that they were pleased with both the care they received and the successful outcome of the treatment.

The clinical photographs were captured using a Samsung Galaxy S24 Ultra, with the native camera app set to pro mode, using the 108MP primary sensor, manual focus, ISO settings between 100 and 400 and adequate lighting to ensure high-resolution and detailed clinical documentation. The images were evaluated in a controlled setting using high-resolution monitors. The documentation was done for educational purposes and to record healing and follow-up progress.

## 3. Discussion

The etiological factor in the discussed case could be due to unfavourable sequelae of dental trauma, a history of prolonged splinting and a poorly endodontically managed tooth [1, 6]. The organic precementum and predentin enveloping the root externally and internally, respectively, are responsible for resisting resorption of the root. These protective layers may be lost or altered, either due to direct trauma or due to the inflammatory response that follows trauma or chronic infection, resulting in resorption cavities on the root surface. Resistance of the unmineralised predentin layer to resorption is conferred by the presence of inhibitors of macrophage propagation. Clastic cells do not resorb or adhere to the unmineralised matrix. RGD peptides are the mediators of osteoclast binding, and these are present only on mineralised surfaces [1, 3, 11, 12].

Root resorption could be internal or external. External root resorption can be further divided as external cervical resorption (ECR), surface resorption, transient apical breakdown and EIRR. EIRR is currently referred to as IRR [9, 13].

In the present case, maxillary central and lateral incisors were affected by different types of root resorption. The diagnosis was invasive cervical root resorption with pulp canal obliteration (PCO), internal inflammatory resorption, IRR and blunderbuss canal in relation to Tooth Numbers 12, 21, 11 and 22, respectively, as a sequelae of trauma and incomplete management.

These types of inflammatory root resorption may be dependent on the following factors:

1. Management after the initial injury, such as improper splinting and ineffective endodontic management.
2. Injury to precementum, periodontal ligament (PDL) and/or predentin.
3. Exposure of dentinal tubules.
4. Age of the patient.
5. Microbial diffusion between infected pulp, PDL space and areas of resorption on the root [14, 15].

All the above mentioned factors were present in this case. There was a history of trauma resulting in the fracture of Tooth Numbers 21 and 22, improper splinting of central and lateral incisors which were luxated at the time and incomplete root canal treatment of Tooth Number 11.

Splinting is recommended to stabilise traumatised teeth and permit healing. However, studies have shown that longer periods of splinting and the use of rigid splints have been associated with an increased risk of occurrence of complications. Instead, flexible splints for shorter periods of time are recommended. This will permit physiological mobility of the teeth, which will act as a stimulus for regeneration and healing of the traumatised tooth [16, 17]. The recommended duration of splinting following luxation for PDL healing is 2–4 weeks, and the duration may be increased to 6–8 weeks when it is associated with alveolar fracture [9].

IRR is characterised by resorption cavities involving the external surface of the root with chronic apical periodontitis. The initiation phase of IRR is characterised by the rupture of the PDL and damage to the outermost cemental layer, which may occur due to trauma, infection or mechanical force on the PDL (orthodontic treatment). Due to injury, there is a release of proinflammatory cytokines that recruit odontoclasts to the site of injury, resulting in the resorption process. Bacteria from necrotic pulp may traverse the dentinal tubules to the area of resorption, leading to further progression of resorption. In the progressive phase, the progression of the resorption occurs due to bacterial stimulation of clastic cells and a viable blood supply within the root canal [5, 11, 18]. Following trauma, pulpal necrosis does not occur completely; portions of the pulp still remain vital. Remaining vital pulp provides nutrients that sustain clastic cells [18].

In this case, Tooth Number 12 was also diagnosed with Class 4 Heithersay ECR and PCO, where the resorptive defect extended beyond the coronal third of root dentin.

ECR is a rare and aggressive form of IRR. ECR involves the cervical aspect of the tooth immediately below the epithelial attachment, and it progresses towards the dentin of the root. This form shares a similar pathophysiology as other forms of root resorption. The cervical region of the tooth is characterised by a lack of precementum as compared to the middle and apical thirds of the root. This could be a factor in its susceptibility to resorption [10, 19].

As ECR progresses inwards, the innermost organic predentin layer is usually not involved; instead, it progresses circumferentially and apico-coronally. ECR is characterised by excessive destruction of cementum and dentin with or without pulpal involvement. Simultaneous inflammation caused by bacteria in the periodontal pocket promotes and perpetuates the resorption process [20].

The tooth affected by ECR also presented with a calcified root canal space. PCO is characterised by a progressive reduction in the size of the pulp chamber, and this may result in partial or complete obliteration of the pulp canal space. Histologically, it is characterised by gradual, excessive deposition of reparative dentin within the pulp canal space. It is a form of calcific metamorphosis of the pulp and is a physiological process. PCO is associated with dental trauma and is seen more often in luxation injuries affecting immature teeth [21, 22].

Luxation of a tooth might result in transient disruption of its blood supply. In the area of ischaemia, the odontoblasts get replaced by undifferentiated mesenchymal cells, and these cells deposit reparative dentin in an uncontrolled manner. Narrowing of capillaries due to deposition of calcium may also occur. PCO is also characterised by injury to the neurovascular supply of the tooth. There may be an uncontrolled sympathetic nervous response due to traumatic injury. Another theory states that the bleeding in the canal due to trauma may act as a nidus for calcification, which eventually results in complete obliteration. The type of splinting material and the degree of repositioning of luxated teeth can also affect the occurrence of PCO [23–25].

Internal inflammatory root resorption initiation depends on injury to the predentin layer and odontoblastic layer, followed by exposure of mineralised dentin. Due to localised pulpal inflammation after trauma, pulp tissue expresses RANKL, which is part of the osteoprotegerin (OPG)/RANK/RANKL system that has a role in the differentiation of clastic cells to cause resorption of the mineralised tissue. Pulpal inflammation is characterised by hyperaemia, which results in an increase in the oxygen tension and a reduction of pH. This also plays a role in the recruitment of clastic cells. Resorption of the inner surface of the root is followed by metaplasia of the connective tissue, and there is the formation of granulation tissue in the resorption defects [26, 27].

Pulp necrosis and ischaemia in an immature tooth might also arrest the root development, resulting in a blunderbuss canal with a wide open apex, thin dentinal walls and resorption defects as seen in Tooth Number 22 in the present case. Endodontic treatment is challenging due to the wide open apex and fragile dentinal walls. The goal of the treatment in such cases should be to halt the resorptive process, form an apical barrier and reinforce the root structure to prolong the survival of the tooth [28, 29].

Prompt detection and diagnosis are crucial factors in determining the clinical outcome of treatment of such cases. The use of advanced diagnostic aids such as CBCT aids in the accurate diagnosis of different types of root resorption as it provides a 3D image of the tooth, the lesion and its surrounding structures. The extent of the lesion can be better viewed in a CBCT image rather than just an intraoral periapical radiograph [30, 31].

The objective of the treatment strategy adopted in the present case was to remove necrotic pulp and to disinfect the root canal, including eliminating any remaining vital pulp tissue sustaining the resorption process. Placement of the rubber dam was avoided, as the application of clamps would have exerted additional mechanical stress on the traumatised teeth, potentially exacerbating the mobility of the teeth. Although the teeth presented with mobility, there was no occlusal interference with the opposing dentition; hence, occlusal reduction was not performed.

In Tooth Number 12 with invasive cervical root resorption and obliterated root canal, endodontic treatment was challenging, so the cervical defect was surgically exposed, debrided and restored using MTA. Tooth Numbers 11 and 21 affected by external and internal root resorption underwent root canal treatment. Sodium hypochlorite, saline and chlorhexidine were used to irrigate the root canals, and calcium hydroxide was used as the intracanal medicament. MTA was used as the obturating material in Tooth Numbers 11 and 21. In Tooth Number 22 with a blunderbuss canal and wide open apex, endodontic treatment and apexification were performed using Biodentine.

Warm vertical compaction of gutta-percha and thermoplasticised canal filling techniques have also been shown to be more effective in sealing the resorption cavities [32]. However, bioactive calcium silicate–based materials like MTA and Biodentine have also been used as root canal filling materials [33, 34].

The reason for selecting these materials to obturate the entire root canal is that there were resorption defects along the length of the roots. These materials have the potential to induce hard tissue. They have the property of gradual deposition of hydroxyapatite, thus strengthening the weakened root canal. These materials can also regenerate damaged root cementum and PDL. MTA can release bone morphogenic protein-2, transforming growth factor beta-1 and interleukins. Biodentine also has immunomodulatory properties; it can suppress proinflammatory cytokines and can augment anti-inflammatory cytokines [35–37].

Another important feature is their alkalinity and sustained calcium release. These materials can also reduce the recurrence of the resorption process after treatment. Calcium hydroxide has the property to increase OPG expression, upregulate the OPG/RANKL ratio and prevent the formation of clastic cells [27, 38, 39].

Prognosis of teeth affected by root resorption depends on factors like the age of the patient, medical history, degree of resorption, type of resorption, location of the resorption defect, type of intracanal medicament and obturating material used in the treatment. In this case, the tooth with internal root resorption has a comparatively better prognosis as compared to others with IRR and cervical root resorption. Endodontic treatment followed by reinforcement of the root canal usually ceases the resorptive process in internal root resorption, but in cases of IRR, the resorption process depends not only on factors involving the pulp but also on the external periodontium [40, 41].

The findings of the discussed case suggest the use of bioceramics such as MTA and Biodentine as root canal filling materials for the management of IRR and have promoted the healing and repair of this condition. Early prompt diagnosis, debridement of the necrotic resorptive tissue, obturating and sealing the defect generally lead to successful clinical outcomes. Teeth affected by root resorption may have unpredictable outcomes. However, the main objective of the treatment is to prolong the survival of the affected teeth by halting the resorption process until the child is old enough, as other options of prosthodontic rehabilitation could be explored.

The limitation of this case is that if there is progression of the root resorption process in the future, re-treatment would be challenging as the entire root canal of the affected teeth was obturated using bioceramic materials.

## 4. Conclusion

Management of root resorption is challenging as the prognosis and outcomes are unpredictable. Attempts should be made to prevent or, in certain cases, delay the inevitable external resorption. Obturating or filling the entire length of the canals and the resorption defects with bioceramics or calcium silicates seems to have good outcomes. However, further studies with long-term follow-ups are essential to establish predictable outcomes.

## Data Availability

The data that support the findings of this study are available on request from the corresponding author. The data are not publicly available due to privacy or ethical restrictions.
